# Supplementary material for: Modeling glioblastoma heterogeneity as a dynamic network of cell states
Source: Mol Syst Biol. 2021 Sep 16;17(9):e10105. doi: 10.15252/msb.202010105 (PMC8444284; doi:10.15252/msb.202010105)
Supplement: Supplementary file 6 — Source Data for Figure 5 [file MSB-17-e10105-s004.zip › Figure5A_sourcedata/GSEA_3017/nosigdb_stateC.GseaPreranked.1622795002785/RICHARDS_LIDDELOW_NATURE_2017_A2_REACTIVE_ASTROCYTES.html]

Details for gene set RICHARDS\_LIDDELOW\_NATURE\_2017\_A2\_REACTIVE\_ASTROCYTES[GSEA]

|  || Dataset | state13017 |
| Phenotype | NoPhenotypeAvailable |
| Upregulated in class | na\_neg |
| GeneSet | RICHARDS\_LIDDELOW\_NATURE\_2017\_A2\_REACTIVE\_ASTROCYTES |
| Enrichment Score (ES) | -0.8191516 |
| Normalized Enrichment Score (NES) | -3.1689498 |
| Nominal p-value | 0.0 |
| FDR q-value | 0.0 |
| FWER p-Value | 0.0 |
Table: GSEA Results Summary

  

Fig 1: Enrichment plot: RICHARDS\_LIDDELOW\_NATURE\_2017\_A2\_REACTIVE\_ASTROCYTES      
 Profile of the Running ES Score & Positions of GeneSet Members on the Rank Ordered List

  

| PROBE | GENE SYMBOL | GENE\_TITLE | RANK IN GENE LIST | RANK METRIC SCORE | RUNNING ES | CORE ENRICHMENT || 1 | OSMR |  |  | 292 | -0.274 | -0.4738 | No |
| 2 | TIMP1 |  |  | 399 | -0.320 | -0.6226 | No |
| 3 | LGALS3 |  |  | 471 | -0.374 | -0.7056 | No |
| 4 | IGFBP3 |  |  | 538 | -0.493 | -0.7674 | Yes |
| 5 | TNFRSF12A |  |  | 550 | -0.519 | -0.7318 | Yes |
| 6 | GDF15 |  |  | 554 | -0.531 | -0.6812 | Yes |
| 7 | ANXA2 |  |  | 563 | -0.567 | -0.6354 | Yes |
| 8 | GFAP |  |  | 574 | -0.659 | -0.5834 | Yes |
| 9 | CD44 |  |  | 575 | -0.659 | -0.5142 | Yes |
| 10 | GAP43 |  |  | 576 | -0.660 | -0.4449 | Yes |
| 11 | VIM |  |  | 578 | -0.674 | -0.3758 | Yes |
| 12 | TM4SF1 |  |  | 580 | -0.689 | -0.3052 | Yes |
| 13 | S100A10 |  |  | 582 | -0.691 | -0.2343 | Yes |
| 14 | CTGF |  |  | 588 | -0.782 | -0.1608 | Yes |
| 15 | EMP1 |  |  | 589 | -0.785 | -0.0784 | Yes |
| 16 | VGF |  |  | 592 | -0.845 | 0.0069 | Yes |
Table: GSEA details [plain text format]

  

Fig 2: RICHARDS\_LIDDELOW\_NATURE\_2017\_A2\_REACTIVE\_ASTROCYTES: Random ES distribution      
 Gene set null distribution of ES for **RICHARDS\_LIDDELOW\_NATURE\_2017\_A2\_REACTIVE\_ASTROCYTES**
